# Mucoprotective effect of ellagic acid in 5 fluorouracil-induced intestinal mucositis model

**DOI:** 10.25122/jml-2023-0052

**Published:** 2023-05

**Authors:** Dareen Mahmood AL-hoshary, Munaf Hashim Zalzala

**Affiliations:** 1. Al-Kut Hospital for Gynecology Obstetrics and Pediatrics, Ministry of Health, Baghdad, Iraq; 2. Department of Pharmacology and Toxicology, College of Pharmacy, University of Baghdad, Baghdad, Iraq

**Keywords:** ellagic acid, 5-fluorouracil, intestinal mucositis, oxidative stress, cytokines

## Abstract

Intestinal mucositis (IM) is a common side effect of several anticancer medications, including 5-fluorouracil (5-FU), and can lead to treatment disruptions and compromised outcomes. IM has severe clinical effects such as diarrhea, erythematous mucosal lesions, and the development of ulcers accompanied by excruciating pain. This study aimed to evaluate the mucoprotective effects of ellagic acid on 5-FU-induced IM in mice. Mice were administered normal saline intraperitoneally for six days, followed by intraperitoneal injection of 5-FU for four days at a dose of 50 mg per kilogram. Ellagic acid was orally administered to the mice in groups III and IV in two doses (5 mg and 10 mg), with a one-hour time separation from 5-FU for ten days. At the end of the experiment, small intestine tissue was collected to measure the levels of antioxidant enzymes superoxide dismutase (SOD), glutathione (GSH), malondialdehyde (MDA), and inflammatory cytokines (IL-6, IL-B, TNF) using ELISA assay. Pre-treatment with ellagic acid led to a significant decrease in pro-inflammatory cytokines and improved antioxidant enzyme levels compared to the 5-FU group. Histopathological analysis demonstrated the mucoprotective effect of ellagic acid against 5-FU-induced intestinal changes, including villi atrophy, damage to stem cells, infiltration of inflammatory cells in the mucosal layer, edema, damage to muscular mucosa, and decreased oxidative stress production, such as MDA. These results suggest that ellagic acid may be a potential candidate for treating IM induced by antineoplastic drugs.

## INTRODUCTION

5-Fluorouracil (5-FU) is a commonly prescribed chemotherapeutic agent for various cancer types, including colon, breast, and head and neck. One of the major side effects of 5-FU treatment is intestinal mucositis, characterized by inflammation and damage to the lining of the small intestine. Intestinal mucositis can lead to various symptoms, including diarrhea, abdominal pain, and weight loss [1]. This drug can cause changes in the small intestine mucosa, which occur in five stages: chemotherapeutic-induced generation and up-regulation signals of the messenger, signaling through mediators of inflammation, mucosal ulceration, and amplification in the mucosal damage, finally initiating the healing process [2].

Intestinal mucositis is a common side effect of chemotherapy, affecting between 50% and 80% of patients and leading to clinical symptoms such as ulceration, diarrhea, and abdominal discomfort [3]. Mucositis is caused by chemical or physical damage to the gastrointestinal tract and is commonly caused by chemotherapy, radiation therapy, or surgery. Intestinal mucositis occurs when the intestinal mucosa is exposed to toxic chemicals or physical damage. The development of mucositis is initiated by the initial damage inflicted on cells by radiation and chemotherapy. This damage can occur through direct effects on DNA or indirectly by generating reactive oxygen species. This triggers a complex cascade of enzyme and transcription factor activations, which ultimately result in the upregulation of genes encoding inflammatory cytokines such as interleukin-6 (IL-6), interleukin-1B (IL-1B), and tumor necrosis factor (TNF), leading to tissue damage by targeting the submucosa and basal epithelium [4].

Some factors can increase the risk of developing intestinal mucositis, such as the specific antineoplastic drug, dose, duration of treatment, and frequency. Other factors that affect the severity of mucositis include smoking, age, sex, nutrition status before treatment, and genetic factors. All symptoms of intestinal mucositis occur 5-14 days after receiving 5-fluorouracil [5].

Ellagic acid is a polyphenol found in fruits and vegetables such as berries, pomegranates, raspberries, walnuts, and green tea. Ellagic acid exhibits various biological functions, including antioxidant, anti-inflammatory, and antimicrobial activity. It disrupts reactive oxygen and nitrogen species and acts through the ability to accept electrons and participate in the reaction of antioxidant redox. Ellagic acid also acts indirectly as a scavenger for free radicals to prevent oxidation. In disease models, ellagic acid has shown its ability to decrease oxidative stress markers such as MDA and NO and raise the level of antioxidant enzymes such as SOD, GSH, and CAT. This action is related to its mucosa microbial metabolite, urolithins, and can reduce oxidative injury by regulating apoptosis [6].

The impact of antineoplastic drugs on the small intestine of mice has been extensively studied. Apoptosis is a key factor in developing acute gastrointestinal (GI) damage, and lower doses of chemotherapeutic drugs result in crypt cell apoptosis, shortening the villi over 5-7 days. Full recovery is experienced afterward, indicating that the stem cell compartment has not been permanently damaged. However, in high doses of cytotoxic drugs like 5-fluorouracil, mice will die between 7-12 days due to complications in the small intestine. The resulting damage is so severe that it cannot be resolved by any specific treatment or intervention. This higher dose causes complete removal of the crypt, severe inflammation, and loss of epithelium. The number of crypt cells will decrease, and lymphocyte cells will increase in specific mucosa sites [7].

Ellagic acid helps heal damaged cells in the mucosa. It has anti-oxidant properties and can bind with free radicals that cause cell damage. Free radicals can be caused by chemotherapy and radiation therapy and lead to cell damage, cancer, and other diseases. Ellagic acid also has anti-inflammatory properties and can reduce the severity of symptoms of GI tract damage, including mucositis. It does this by decreasing the production of compounds that cause inflammation in the GI tract, such as prostaglandins. Free radicals caused by chemotherapy and radiation therapy can also damage cells in the GI tract, leading to GI tract damage, including mucositis. Treatment for harmed cells in the GI tract with ellagic acid reduces and subsequently helps heal cell damage and inflammation [6,7].

Ellagic acid (EA) protects against the infiltration of pathogens into the mucosa. It has anti-microbial properties that enhance fighting against infection from pathogens such as *Clostridium difficile* and *E. coli*. Ellagic acid also has anti-fungal properties, which help fight against infection from molds like *Candida albicans* and *Aspergillus niger*. Free radicals caused by chemotherapy and radiation therapy can cause both cell death and tissue damage, leading to infection of the GI tract and other body parts. Ellagic acid protects against this damage, which helps reduce infection in cases of GI tract damage caused by chemo- or radiation therapy [8].

In addition to its anti-inflammatory properties, EA also has other medicinal properties. These include its role in combating lipoperoxidation, decreasing neurogenic pain, and reducing tumor necrosis factor (TNF) levels, which hold significance in conditions like cancer. In addition, EA has shown promising effects in gastrointestinal protection alongside its anti-inflammatory properties [8]. This paper focuses on the role of ellagic acid in protecting against intestinal mucositis and explores safe administration practices.

## MATERIAL AND METHODS

### Materials

Ellagic Acid Powder (90%) extract was obtained from the Galla Chinensis plant ( Xi'an Kono chem co., Ltd., China). Fluorouracil 500mg/10ml ampoules were manufactured by Celon Lab and purchased from Shree Medical Store Pvt Ltd in Delhi. ELISA kits for cytokine analysis were purchased from Jigging in Nanjing, China.

### Animals

Forty Albino male mice weighing 25-42 grams were obtained from the Animal House of the College of Pharmacy at Baghdad University. The mice were housed under normal temperature and humidity and maintained on a regular light/dark cycle. They had access to standard food and water ad libitum [7-9].

### Experimental design

To induce intestinal mucositis, mice were randomly assigned to one of four groups of ten mice each.

- Group I (control group) received 0.9% normal saline with dimethyl sulfoxide (DMSO) orally for ten days; this protocol is commonly used in experimental animals [9,10].
- Group II (model group) was injected intraperitoneally (i.p.) with 5-fluorouracil (50mg/kg) for four consecutive days.
- In Group III (treatment group), mice received high-dose ellagic acid (10mg/kg) by oral gavage for ten days, and from the seventh day, they were injected with (50mg/kg) 5-FU for four consecutive days. The selection of the ellagic acid dose (10 mg/kg) was based on previous studies that have investigated its effects [11,12].
- In Group IV (treatment group), the mice received low-dose ellagic acid (5mg/kg) by oral gavage for ten days, and from the seventh day, they were injected with (50mg/kg) 5-FU for four consecutive days.

### Experimental mucositis induction

All animals in the experiment, except for the vehicle group, were given 5-fluorouracil at a dose of 50mg/kg i.p for four consecutive days, starting from day 7. From day 1 to day 6, all tested groups received an intraperitoneal injection of normal saline [13,14]. In groups III and IV, animals were pretreated with ellagic acid one hour before giving 5-fluorouracil or normal saline [14-16]. The mice were sacrificed under deep anesthesia with diethyl ether on day eleventh.

### Physical manifestations

Animals were kept under close observation by measuring their weight loss and scoring the consistency of their stool (diarrhea scoring). The severity of intestinal mucositis was determined by the degree of diarrhea scoring using the following classification :

- Score (0) - Normal: stool appears normal or is absent.
- Score (1) - Mild: stool is soft and wet.
- Score (2) - Moderate: stool is unformed and wet, with moderate coat staining.
- Score (3)- Severe: stool is watery, with severe coat staining, and may contain bloody watery stool [16].

### Tissue collection

The animals were sacrificed by cervical dislocation under anesthesia at the end of the experiment. The small intestine was removed, and phosphate buffer saline was used for cleaning. A blood sample was collected for CBC (Complete Blood Count) analysis, and a piece of small intestine tissue was kept in 10% neutral buffered formalin for histological examination, and another part of the tissue was homogenized [17].

### Histological examination

A section of the small intestine, specifically the duodenum part, was collected and stained with hematoxylin and eosin (H&E). Approximately 3 cm of the small intestine was washed with 0.9% normal saline, fixed in 10% formalin solution for about one hour, dehydrated in high concentrations of ethanol, and then covered in paraffin wax for 24 hours. A part of about 4-micrometer thickness was cut and fixed on a glass slide for H&E staining. Histological analysis was used to measure villus height and crypt presence or any changes in the mucosal layer affected by 5-fluorouracil and treatment. The score for each sample (five samples for each group) was determined under a light microscope using a calibrated micrometer at various magnifications (4x, 10x, and 40x) [18,19].

### Assessment of anti-oxidant enzymes glutathione (GSH), superoxide dismutase (SOD), and malondialdehyde (MDA) levels

After removing a part of the small intestine (duodenum part) and washing it with a cool buffer saline solution, a homogenizer was used for tissue homogenization. The resulting serum was centrifuged at 5000 rpm for 15 minutes at 4°C, and the supernatant serum was separated using a micropipette and stored at -18°C until the day of analysis of the antioxidant enzyme glutathione (GSH), superoxide dismutase (SOD), and malondialdehyde (MDA) concentrations in the intestinal tissue using ELISA analysis [20]. The competitive enzyme immunoassay technique was employed in this study. According to this method, a combination of (anti-oxidant enzyme) monoclonal antibody and HRB-conjugate, along with the buffer, was incubated with the assay sample and HRP-conjugate on a pre-coated plate for one hour. After the end of the incubation period, the wells were washed with wash buffer five times [8-10].

The substrate was then added to the well and left in the incubation period for 15 minutes in a dark place. The reaction product from the complex had a blue color. Finally, the reaction was stopped by adding a stop solution, so the solution became yellow. As the color increased in intensity, the concentration of anti-oxidant in the sample was high, and this could be measured spectrophotometrically at 450 nm in a microplate reader.

### Assessment of pro-inflammatory cytokines

Pro-inflammatory cytokines (IL-1β, IL-6, and TNF-α) in the intestinal tissue were determined using ELISA assay kits [9].

### Measurement of Complete Blood Count (CBC)

Mice were sacrificed by cervical dislocation, and the blood was collected in an EDTA tube and then kept at 4°C. Blood counts were measured using an SYSMEX XE-2100 Hematology analyzer (Sysmex, Kobe, Japan).

### Mouse body weight variation

We measured the body weight variation of mice every day at 8 AM. The values were presented as a percentage change in weight from the starting weight [21]. Weight Variation % = ((W2 - W1)/W1) x 100 where: W1 is the initial mouse weight. W2 is the weight of the mouse at the time of the study [22].

### Statistical Analysis

The mean and standard deviation were used to express the results. One-way analysis of variance (ANOVA) and Dunnett's test were then used to determine the differences between the groups after determining the normality of the distribution of values for each group [22].

## RESULTS

There was a significant increase in TNF levels in group (I) compared to the vehicle group (p<0.05), as shown in Table 1. In addition, in group (III), which received a high dose of ellagic acid, a significant difference was observed when compared with group (II) (P<0.05). Group IV, which received a low dose of ellagic acid, exhibited a significant elevation in TNF-alpha levels compared to group III (p<0.05).

**Table 1.** Level of TNF-α, GSH, and IL-1β across all groups

Treatment group, N=10	Type of treatment	TNF-α (ng/ml) mean±S.D	GSH (ng/ml) mean±S.D	IL-1β (ng/ml) mean±S.D
I	Normal saline +DMSO	56.28±11.84	6.01±2.13	8.92±1.12
II	5-FLU.	166.84±73.02^*a^	3.01±0.23^a*^	20.04±13.75^*a^
III	Ellagic acid 10mg/kg	49.95±5.20^b^	5.04±0.68^b^	10.16±1.14^b^
IV	Ellagic acid 5mg/kg	65.40±7.79^*c^	3.83±0.32^*^	12.00±1.33^c^

significant difference between the control group (I) and induction group(II) (*) Values with non-identical small letter superscripts (a, b, and c) are considered significantly different P<0.05. N = number of animals in each group. Data are presented as mean± standard error of means (SEM)

According to the data presented in Table 1, a significant difference (p<0.05) in glutathione (GSH) levels between groups was observed. Specifically, there was a significant decrease in GSH levels in group (II) compared to the vehicle group (I). The mean GSH level in group III was approximately similar to that of the vehicle group (I).

The results presented in Table 1 demonstrate a significant increase in IL-1β enzyme levels in mice that received 5-FU in Group II compared to the normal control group I (p<0.05). Moreover, the group of mice that received a high dose of ellagic acid (10 mg/kg) showed no significant difference compared to the normal control group (p>0.05). When comparing Group II, Group III, and Group IV, the intended level of the enzyme decreased after the administration of ellagic acid for ten days before the 5-FU dose. The level of IL-1β in group (III) was lower than in group (IV) (Table 1).

According to the results, the diarrhea score increased in the induction group (group II) after 24 hours following the final dose of 5-fluorouracil injection on day 5. As shown in Table 2, the administration of ellagic acid had a significant effect on stool nature and improved the severity of diarrhea.

**Table 2.** Effect of ellagic acid on stool nature

Treatment group N=10	Type of treatment	Diarrhea scoring Mean ±S.D
I	Normal saline and DMSO	0
II	5-fluorouraci	1.94±1.13^*a^
III	Ellagic acid 10mg/kg	0.12±0.11^b^
IV	Ellagic acid 5mg/kg	1.13±0.88^*^

(*): significant difference compared with the normal control group ( a, b) the significant difference between groups Data are presented as mean± standard error of means (SEM)

There were significant differences observed between group (I), group (II), and between group (II) and group (III) (p<0.05). The induction group experienced severe and bloody diarrhea, especially on day 5. In contrast, the ellagic acid group (Group III) did not show a significant difference compared to the normal control group (Group I) (p>0.05), and there were fewer diarrhea symptoms compared to the induction group (Group II). Group IV had a significant difference compared to the normal control group (Group I) (p<0.05), while there was no difference between Group IV and Group II (p>0.05)

## HISTOPATHOLOGICAL EXAMINATION OF MICE DUODENUM SECTION

**Figure 1.**
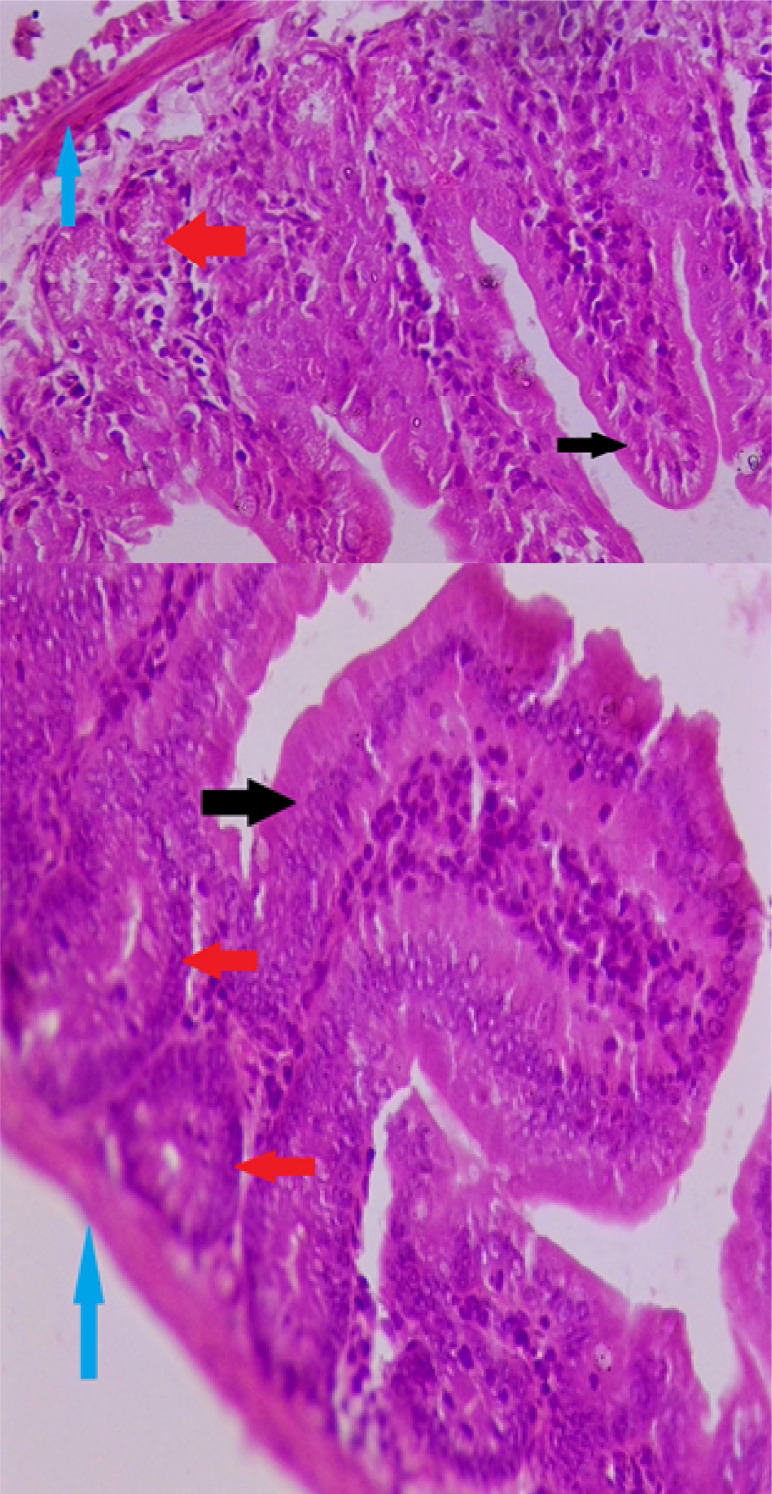
Cross-section shows normal mice duodenum in group (I) **Magnification (100X), staining: Hematoxylin and eosin** **Black arrow:** represents normal intestinal villi without histopathological changes. **Red arrow:** indicates normal mitotic crypt cell. **Blue arrow:** indicates the muscularis layer (thin layer) in the normal case without damage.

The intestinal villi in group I (the normal saline group) had a normal structure, no inflammatory cell infiltration, no dysplastic cell change, no edema or lymphocyte cell infiltration (Figure 1).

**Figure 2.**
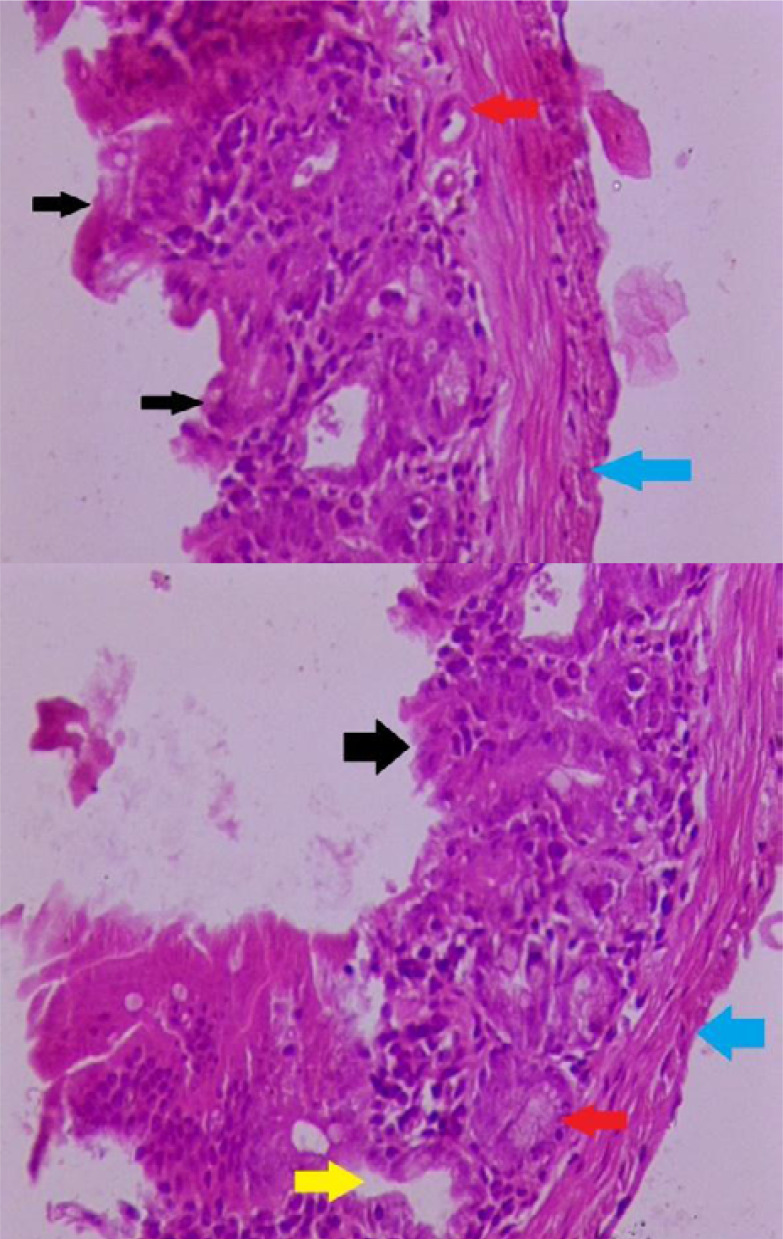
Cross-section of mice duodenum in group (II) **Magnification (100X), staining: Haematoxylin and eosin** **Black arrow:** indicates villi atrophy and shortening **Red arrow:** represents a loss in the number of crypt cells **Blue arrow:** swelling and inflammation of muscularis layer (thick layer) **Yellow arrow:** edema in the lamina propria.

In Group II, the administration of 5-fluorouracil (5-FU) via intraperitoneal injection induced significant histopathological changes in the small intestine tissue. This is evident in the villi atrophy and significant shorting in the length of it, edema in the mucosa and muscularis layer, decrease in crypt cell number, and inflammatory cell infiltration in the lamina propria, as shown in Figure 2.

**Figure 3.**
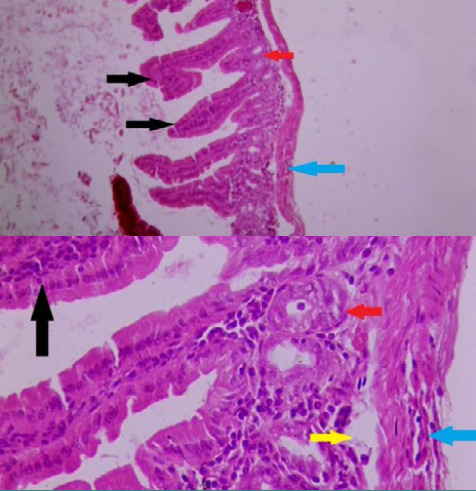
Cross-section of mice duodenum in group (III) **Magnification:100x and 400x, staining: hematoxylin and eosin** **Black arrow:** villi appear in normal shape with less damage compared to group (II) **Red arrow:** decreased number of crypt cells compared to the normal control group. **Blue arrow:** presence of some fluid in the muscularis layer **Yellow arrow:** a sign of edema in the mucosa layer.

In group III (ellagic acid 10mg/kg), the histopathological examination showed reduced morphological changes within the small intestine. Specifically, the specialized intestinal villi appeared closer to their normal shape with less damage. Additionally, there was a decrease in the number of inflammatory cells (Figure 3).

**Figure 4.**
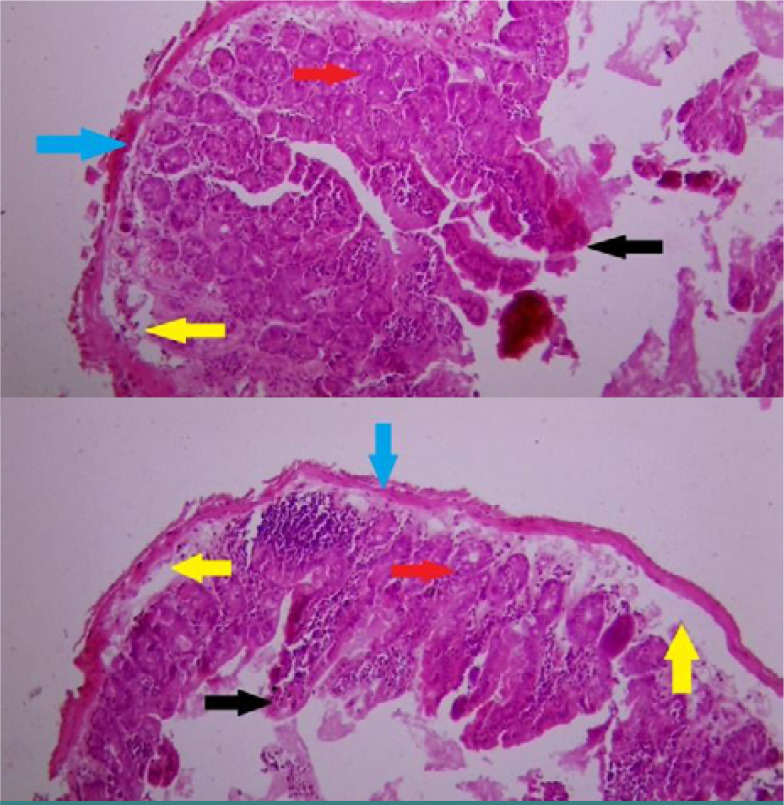
CHistological changes in duodenum tissue of mice in group (IV) treated with ellagic acid at a dose of 5mg/kg. **Magnification:100x and 400x, staining: hematoxylin and eosin** **Black mark:** indicates shortening of the intestinal villi and disruption **Red mark:** shows the accumulation of inflammatory cells in the crypt cells, with loss of crypt parallelism. **Yellow mark** indicates fluid accumulation in the mucosa and muscularis layers of the duodenum tissue. **Blue arrow:** indicates a thin muscularis layer as in the normal control group.

In Group IV, the mice that received a dose of ellagic acid at 5mg/kg exhibited a noticeable shortening of the villi in the duodenum tissue compared to the model control group. Furthermore, the presence of chronic inflammatory cells in the mucosa layer was reduced in Group IV. No dysplastic changes were observed in the examined tissue sections (Figure 4).

## DISCUSSION

Intestinal mucositis is severe inflammation and ulceration of the mucous membranes enclosing the digestive tract caused by cancer treatments such as chemotherapy and radiotherapy. Cytotoxic chemotherapy can alter the functional and structural architecture of the GIT. After chemotherapy, heartburn, abdominal discomfort, diarrhea, digestive problems, bloating, and nausea are common gastrointestinal symptoms. These symptoms occur due to the damage done by cytotoxic drugs [21].

The administration of 5-fluorouracil (5-FU) and its metabolite fluorouridine triphosphate (FUTP) can induce direct harm to the intestinal epithelial cells. This damage can result in DNA impairment and the production of reactive oxygen species due to oxidative stress. This drug can activate multiple signaling pathways, including nuclear factor-kappa B (NF-kB), which can increase the levels of inflammatory cytokines such as TNF and (IL-1B, as well as the expression of inflammatory mediator factors like prostaglandin E2 (PGE2) [22].

Several factors, including the dose and duration of treatment, disease type, and apoptotic factors, can contribute to the development of intestinal mucositis caused by 5-FU. Various mechanisms have been proposed to explain how this drug damages the small intestine, such as oxidative and nitrosative stress and upregulation of inflammatory mediators that promote apoptosis, which, in turn, exacerbates inflammation and tissue damage. These processes are all essential for the pathogenesis of 5-FU-induced small intestine injury in mice [23,24].

The pathophysiology of intestinal mucositis involves changes in the height of the intestinal villi, dimensions of crypts, peroxidation, apoptosis, inflammatory cells, intestinal cell proliferation, and gut microbiome composition. A five-stage model of chemotherapeutic-induced mucositis pathogenesis has been proposed, including occurrence, upregulation, message creation, triggering and amplification, ulceration and inflammatory process, and finally, healing [25].

Increasing the dosage of ellagic acid may lead to a decrease in white blood cell count and counter its mucoprotective effects. As explained above, ellagic acid has many properties useful against colonic damage caused by chemotherapy and radiation therapy. Unfortunately, too much ellagic acid can interfere with white blood cell function instead of helping them do their function better. White blood cells are responsible for fighting off invading pathogens and reducing inflammation throughout the body, so they are very important components when dealing with chemo- or radiation therapy's side effects on the GI tract. When excess white blood cells are present in the body and are engaged in fighting the side effects of chemotherapy or radiation on the GI tract, they may be unable to perform their primary functions effectively. This scenario can potentially create more challenges than solutions in some cases, as the overabundance of white blood cells may attempt to handle multiple tasks concurrently, compromising their efficacy. Instead of dispersing their efforts across numerous functions with limited efficacy, white blood cells should prioritize tasks that align best with their current numbers [26].

According to the study, 5 dosages of intraperitoneal injection ( 50 mg/kg - 100 mg/kg) per day corresponded to the above criteria, and dosages less or more than this concentration might result in either inadequate mucosal injury or a high rate of mortality [12,27].

Damage to the epithelial cells of the small intestine can lead to increased intestinal wall permeability, which allows bacteria and toxic metabolites to penetrate the compromised mucosal barrier, contributing to the development and progression of intestinal mucositis. As a result, improving the function of the bowel mucosal barrier may be a promising therapeutic target for reducing intestinal inflammation[27].

## CONCLUSION

The findings of this study suggest that ellagic acid treatment effectively reduces both the severity and incidence of diarrhea among the experimental subjects. This indicates that ellagic acid can potentially alleviate gastrointestinal distress associated with mucositis, with a specific focus on addressing diarrhea symptoms. These results highlight the favourable role of ellagic acid as a therapeutic intervention for managing mucositis-related gastrointestinal symptoms, particularly diarrhea. Our findings on the mucoprotective effects of ellagic acid have the potential to inform the reduction of the harmful effects of anticancer agents on the intestinal mucosa and consider ellagic acid as a promising therapeutic candidate.
